# Impact of front-of-pack labels on the perceived healthfulness of a sweetened fruit drink: a randomised experiment in five countries

**DOI:** 10.1017/S1368980021004535

**Published:** 2022-04

**Authors:** Alejandra Jáuregui, Christine M White, Lana Vanderlee, Marissa G Hall, Alejandra Contreras-Manzano, Claudia Nieto, Gary Sacks, James F Thrasher, David Hammond, Simón Barquera

**Affiliations:** 1 Nutrition and Health Research Center, National Institute of Public Health, Av. Universidad 655 Col. Santa María Ahuacatitlán, CP, Cuernavaca, MR 62100, México; 2 School of Public Health Sciences, University of Waterloo, Waterloo, Ontario, Canada; 3 École de Nutrition, Centre NUTRISS (nutrition, santé et société), Université Laval, Québec, Québec, Canada; 4 Department of Health Behavior, Gillings School of Global Public Health, Carolina Population Center, Lineberger Comprehensive Cancer Center, University of North Carolina, Chapel Hill, NC, USA; 5 Global Obesity Centre, Deakin University, Burwood, Victoria, Australia; 6 Arnold School of Public Health, University of South Carolina, Columbia, SC, USA; 7 Center for Population Health Research, National Institute of Public Health, Cuernavaca, MR, México

**Keywords:** Food labelling, Perceived healthiness, Consumer perception, International study

## Abstract

**Objective:**

Front-of-pack (FOP) nutrition labelling is a globally recommended strategy to encourage healthier food choices. We evaluated the effect of FOP labels on the perceived healthfulness of a sweetened fruit drink in an international sample of adult consumers.

**Design:**

Six-arm randomised controlled experiment to examine the impact of FOP labels (no label control, Guideline Daily Amounts (GDA), Multiple Traffic Lights, the Health Star Ratings (HSR), Health Warning Labels, and ‘High-in’ Warning Labels (HIWL)) on the perceived healthfulness of the drink. Linear regression models by country examined healthfulness perceptions on FOP nutrition labels, testing for interactions by demographic characteristics.

**Setting:**

Online survey in 2018 among participants from Australia, Canada, Mexico, United Kingdom (UK) and United States.

**Participants:**

Adults (≥18 years, *n* 22 140).

**Results:**

Compared with control, HIWL had the greatest impact in lowering perceived healthfulness (*β* from −0·62 to −1·71) across all countries. The HIWL and the HSR had a similar effect in Australia. Other labels were effective in decreasing the perceived healthfulness of the drink within some countries only, but to a lower extent. The GDA did not reduce perceived healthfulness in most countries. In the UK, the effect of HIWL differed by age group, with greater impact among older participants (> 40 years). There were no other variations across key demographic characteristics.

**Conclusions:**

HIWL, which communicates clear, non-quantitative messages about high levels of nutrients of concern, demonstrated the greatest efficacy to decrease the perceived healthfulness of a sweetened fruit drink across countries. This effect was similar across demographic characteristics.

Front-of-pack (FOP) nutrition labelling is a policy intervention to address the growing global burden of diet-related non-communicable diseases^(1)^. FOP labels aim to provide simplified or interpretative information on the nutritional quality or critical nutrient quantity (nutrients that may pose a substantial public health concern due to overconsumption, such as saturated fat, sugar and Na)^(2)^ of a food product or about the health consequences of consuming nutrients or products, to help consumers make inferences about the healthfulness of the product and support more nutritious choices^(3,4)^. However, more research is needed to inform countries’ decisions about which FOP system to use^(4)^, and to assess potential differential effects among sub-groups^(4,5)^.

Various FOP labelling systems are implemented or being considered by governments globally^(6)^. Labelling systems can be classified as interpretive (i.e. providing nutrition information as guidance rather than specific facts) or reductive (i.e. showing information only, with no specific judgement, opinion or recommendation), as summary indicators (i.e. providing an overall qualification of the product healthfulness) or nutrient-specific systems (i.e. providing nutrition information for a set of nutrients)^(6)^. Some of the most commonly employed systems include the Guideline Daily Amounts (GDA), Multiple Traffic Lights (MTL), Health Star Rating (HSR), ‘High-in’ Warning Labels (HIWL) and Health Warning Labels (HWL) (Fig. 1). GDA are a reductive approach with no interpretative information developed by the food industry, which provide information about the nutrient amounts within a food and its contribution to adult recommended daily intake. This labelling format is voluntarily implemented by the food industry in several countries, including Canada and the United States (US) and was mandatory in Mexico from 2014 to 2020^(6)^, when they were replaced by warning labels. MTL are interpretive nutrient-specific labels which provide similar information as GDA, but colour code each nutrient in order to communicate whether the product contains relatively low (green), average (yellow) or high (red) levels of critical nutrients. MTL have been implemented voluntarily in the United Kingdom (UK) since 2013, and approximately two-thirds of products in the UK carried the MTL in 2016^(6,7)^. MTL have been implemented similarly in other countries, including Ecuador^(8)^, Sri Lanka^(9)^ and Iran^(10)^. The HSR, an interpretive summary indicator endorsed by the governments of Australia and New Zealand for voluntary implementation since 2014, synthesises positive and negative nutrient information into a single dimension of healthfulness, rating the overall nutritional quality of the product from 0·5 to 5 stars^(11)^. In 2017, the HSR system appeared on 28 % of foods^(6,12)^. HIWL are interpretive nutrient-specific labels that show warning symbols (often octagonal) on food packages if energy and key nutrients (sugar, saturated fat and Na) exceed established thresholds and were first introduced in Chile in 2015^(6)^. From an international regulatory and trade perspective, HIWL have been identified as a feasible mandatory system to implement^(13)^, and legislations for mandatory HIWL have been implemented in Israel, Uruguay, Peru and Mexico^(6,14)^, and proposed or approved in Brazil^(15)^, Argentina^(16)^ and Canada^(17)^. Lastly, an interpretive nutrient-specific text-only HWL for sugar-sweetened beverage advertisements has been enacted in San Francisco, US but is being challenged in court and has also been proposed in seven US states^(18)^.

**Fig. 1:**
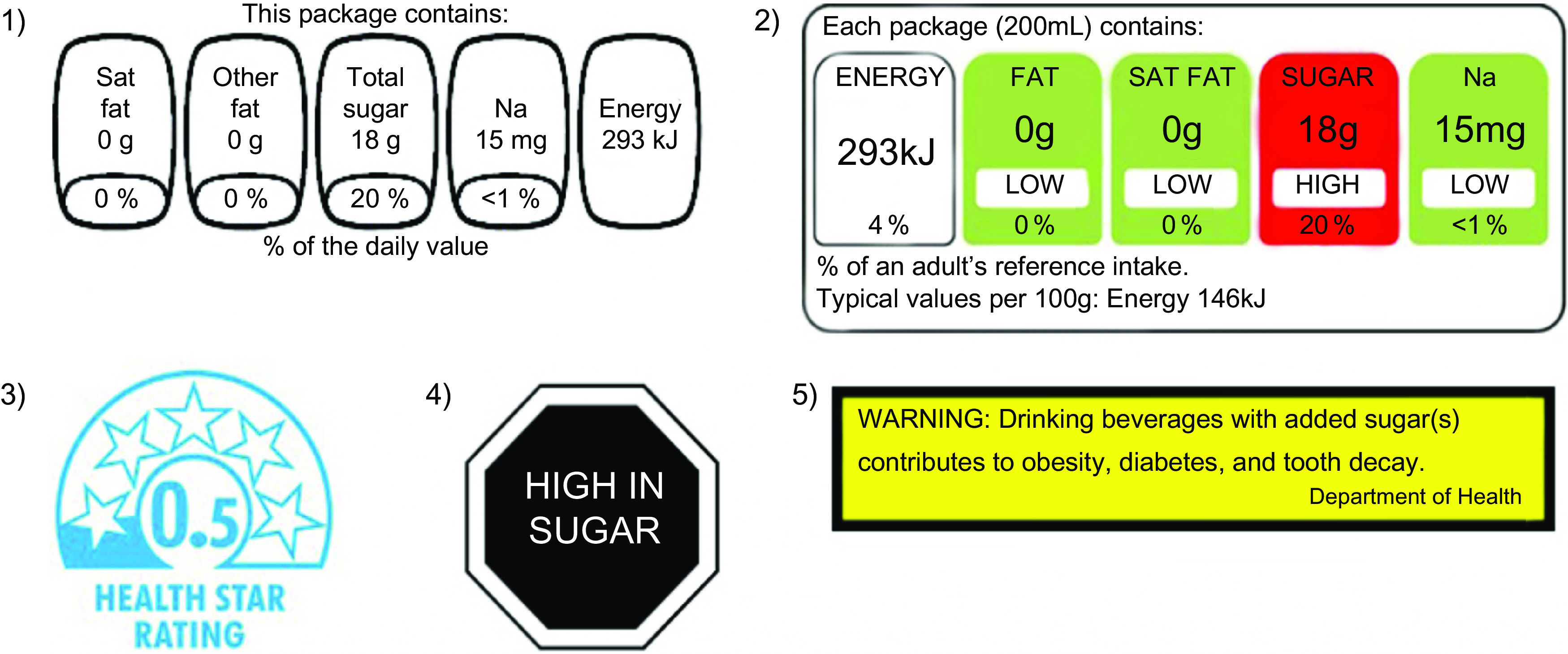
Front-of-pack labels shown on product during experiment. (1) Guideline Daily Amounts (GDA), (2) Multiple Traffic Lights (MTL), (3) Health Star Rating (HSR), (4) ‘High-in’ Warning Labels (HIWL) and (5) Health Warning Labels (HWL)

FOP labels are theorised to shape purchasing and consumption behaviours through several mechanisms. Once noticed by the consumer, FOP labels may change the motivation to consume food products by modifying the way in which they are perceived^(19)^. For example, highlighting the high content of nutrients of public health concern may decrease perceived healthfulness of a product previously misperceived as healthy (e.g. sweetened yogurt or sugary fruit drinks). Indeed real-life experiments suggest that changes in the perceived healthfulness of food products may influence consumption.^(20,21)^ Studies examining the effects of FOP labels on perceived healthfulness suggest that nutrient-specific labels (e.g. MTL, HIWL) and the HSR may be more effective in leading to lower ratings of unhealthy foods compared with GDA^(5,22–24)^. A meta-analysis of experimental studies found that sugar-sweetened beverage warnings (including both HIWL and HWL) successfully lowered healthfulness perceptions compared with control conditions^(25)^. Finally, a recent scoping review of experimental studies of HIWL reported that these labels led to lower perceived healthfulness of products compared with control conditions or other labelling formats (i.e. GDA)^(26)^.

However, a key question is whether FOP label effects are generalisable across countries. Previous international studies exploring country differences on labelling outcomes (e.g. perceived product healthfulness, label perceptions) have found inconclusive results, with some reporting differences across countries^(27–29)^, whereas others have not^(30–32)^. To date, most of these international studies have been conducted in Europe^(27,29,31)^, with less representation of other regions in the world^(28,33)^. Additionally, a call has been made to focus research on the reach of FOP labels’ effects across sub-groups of consumers such as those with varying levels of nutrition knowledge, or among low-income populations^(5,26)^.

The objective of this study was to test the effect of different kinds of FOP labels (GDA, MTL, HSR, HWL and HIWL) on the perceived product healthfulness in an international sample of adult consumers, including evaluation of differences by socio-demographic characteristics and country.

## Methods

### Study design and recruitment

A six-arm, unblinded online randomised experiment was conducted as part of the broader 2018 International Food Policy Study (IFPS), a cross-sectional survey of adults aged ≥18 years (*n* 22 824) from Australia, Canada, Mexico, the UK and the US, who completed an online survey in 2018. The IFPS assesses seven primary policy domains including price/taxation, food packaging and labelling, retail food policies, food marketing, nutritional labelling in restaurants, nutrition information and education, and food guide/dietary recommendations. For the present study, we analysed responses to one single question regarding the perceived healthfulness of a fruit drink labelled with differing FOP labels. The countries represent different policy approaches to FOP labels, as outlined above.

Approximately 2·9 % of participants (*n* 684) were excluded due to missing data in the outcome (*n* 634) or a technical glitch in the survey platform making participants view all experimental conditions on the screen (*n* 50), leaving 22 140 participants for analyses (Australia = 3964; Canada = 4311; Mexico = 4057; UK = 5290; US = 4518). Small differences between included and excluded participants were observed (*P* < 0·01) (online supplementary material, Supplemental Table 2). Missing data in the outcome across label conditions ranged from 1·8 to 3·7 % (*P* < 0·001) (online supplementary material, Supplemental Table 3).

Participants were recruited through the Nielsen Consumer Insights Global Panel and their partners’ panels using both probability and non-probability sampling methods. Random samples were drawn from online panels in each country, stratified by age and sex proportional to the general population in each country. Respondents provided consent prior to completing the survey and received remuneration in accordance with their panel’s usual incentive structure (e.g. points-based or monetary rewards). Surveys were conducted in English in Australia and the UK; Spanish in Mexico; English or French in Canada; and English or Spanish in the US.

### Participants’ allocation and intervention

Using a central computer system, participants were randomly assigned to view on screen one of six images (6·5 cm × 13 cm) of a sweetened fruit drink with differing labelling: no label (control), GDA, MTL, HSR, HWL or HIWL (Fig. 1). These labelling systems were selected as they are either implemented or being considered as a policy option in the five IFPS countries. Researchers were blinded to the assigned intervention, but blinding of participants was not possible given the nature of the intervention.

**Fig. 2:**
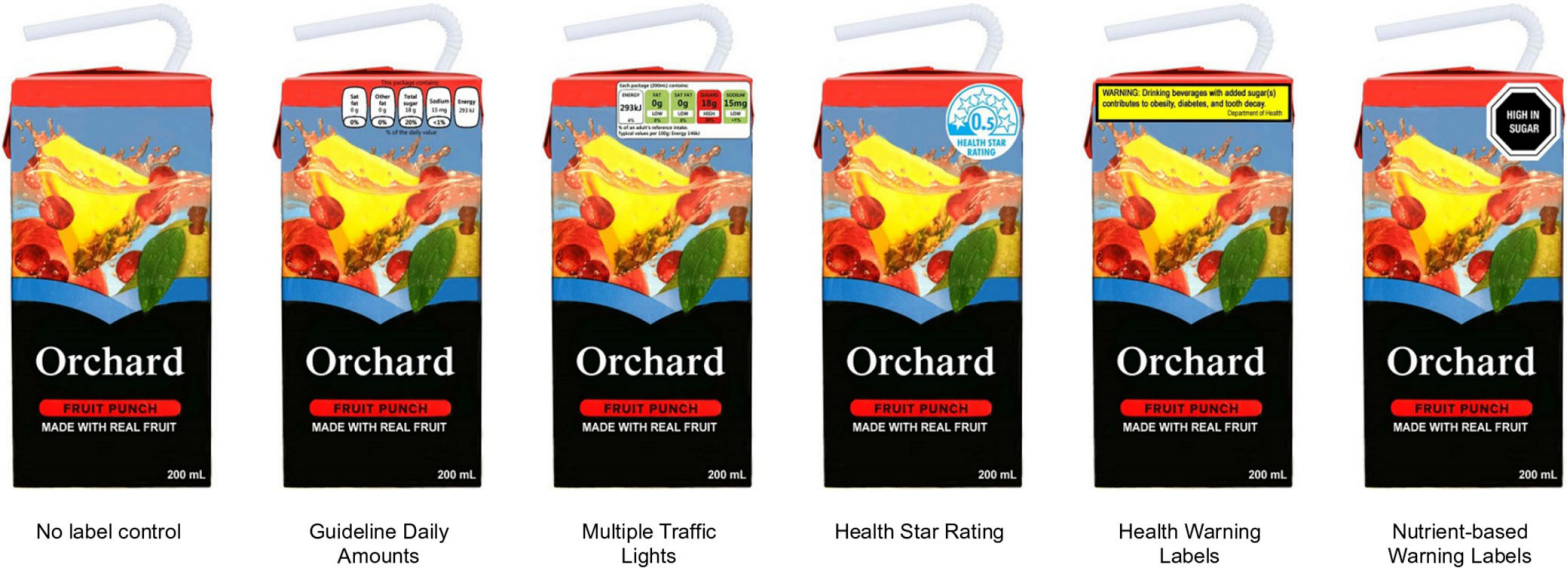
Images with front-of-package labels displayed on screen during experimental task. Note: each participant was only shown one image, corresponding to their assigned condition. Images above were shown in Australia surveys; product and labels varied slightly by country (see online supplementary material, Supplemental Fig. 1)

FOP labels were displayed in the upper right corner of the front of the pack (Fig. 2 and online supplementary material, Supplemental Fig. 1). A sweetened fruit drink was utilised as the test product because processed fruit drinks are considered to be sugar-sweetened beverages and are commonly misperceived as healthy^(34,35)^, despite their high added sugar content and high contribution to energy intake^(36)^. The sweetened fruit drink box image was modelled after a popular drink package to appear authentic, but digitally altered to display fictitious brand names. Package text language and units of measures were altered to match typical product packaging in each country.

### Nutritional criteria for labelling systems

Online supplementary material, Supplemental Table 1 shows the nutrition information used in the development of the FOP labels for the sweetened fruit drink. The nutrient content in the MTL condition was classified according to criteria set out by EU Regulation No. 1169/2011 (e.g. sugar content >13·5 g/portion coded red with ‘high’ text)^(37)^. The online HSR Calculator was utilised to calculate an HSR of 0·5 stars^(38)^. The ‘High in Sugar‘ warning label was applied based on a cut-off of 18 g of sugar per serving size^(39)^ or 5 g/100 ml, as per criteria used in Chile^(40)^.

### Outcome

Participants were asked ‘In your opinion, is this product…’ with nine response options: (1) very unhealthy, (2) unhealthy, (3) a little unhealthy, (4) neither unhealthy nor healthy,(5) a little healthy, (6) healthy, (7) very healthy, (8) don’t know and (9) refuse to answer. Those answering options (8) or (9) (*n* 634, 2·9 %) were considered as missing and excluded from analyses.

### Covariates

Demographic information was assessed using survey measures^(41)^ from population-level surveys within each country^(42–46)^. Variables were recoded and harmonised for comparison across countries and included gender, age group, education, ethnicity, income adequacy^(47)^, self-reported nutrition knowledge, household responsibility for food shopping, frequency of using a nutrition facts table and self-reported BMI (see Table 1). Further details on the IFPS are available elsewhere^(48)^.

**Table 1:** Demographic characteristics in the total sample and by experimental condition (weighted)

	Total (*n* 22 140)		Control (no label) (*n* 3612)		Guideline Daily Amounts (GDA) (*n* 3647)		Multiple Traffic Lights (MTL) (*n* 3711)		Health Star Rating (HSR) (*n* 3735)		Health Warning Label (HWL) (*n* 3736)		‘High-in’ Warning Label (HIWL) (*n* 3699)		*P* ^*^
	%	99 % CI	%	99 % CI	%	99 % CI	%	99 % CI	%	99 % CI	%	99 % CI	%	99 % CI	
Country															
Australia	17·9	17·1, 18·7	18·0	16·2, 20·0	18·5	16·7, 20·5	18·0	16·2, 20·0	18·5	16·7, 20·5	17·3	15·5, 19·3	17·2	15·4, 19·1	0·687
Canada	19·5	18·7, 20·3	19·2	17·3, 21·3	19·6	17·6, 21·7	19·2	17·3, 21·3	19·5	17·5, 21·5	19·9	17·9, 22·0	19·5	17·5, 21·6	
Mexico	18·3	17·5, 19·2	16·8	15·0, 18·8	18·2	16·3, 20·3	18·9	17·0, 21·0	18·2	16·3, 20·2	19·0	17·1, 21·1	18·8	16·9, 20·9	
United Kingdom	23·9	23·0, 24·8	24·9	22·7, 27·3	23·5	21·4, 25·7	24·3	22·2, 26·5	23·0	20·9, 25·2	23·4	21·3, 25·6	24·3	22·2, 26·4	
United States	20·4	19·6, 21·3	21·0	18·9, 23·3	20·2	18·3, 22·3	19·6	17·7, 21·8	20·8	18·8, 23·0	20·4	18·3, 22·5	20·3	18·3, 22·4	
Gender															
Male	48·5	47·5, 49·6	48·6	46·0, 51·1	48·2	45·6, 50·8	49·2	46·7, 51·8	49·0	46·5, 51·5	49·4	46·9, 52·0	46·7	44·2, 49·2	0·389
Female	51·5	50·4, 52·5	51·4	48·9, 54·0	51·8	49·2, 54·3	50·8	48·2, 53·3	51·0	48·4, 53·5	50·6	48·0, 53·1	53·3	50·8, 55·8	
Ethnicity^†^															
Majority group	80·0	79·0, 80·8	79·7	77·3, 81·9	79·5	77·2, 81·7	79·9	77·5, 82·0	79·4	77·1, 81·5	81·0	78·8, 83·1	80·2	77·9, 82·3	0·975
Minority group	20·0	19·2, 21·0	20·3	18·1, 22·7	20·5	18·3, 22·8	20·1	18·0, 22·4	20·6	18·5, 22·9	19·0	16·9, 21·2	19·8	17·7, 22·1	
Age group															
18–29	22·5	21·6, 23·4	22·5	20·3, 24·7	21·3	19·2, 23·5	23·4	21·5, 25·9	22·6	20·5, 24·7	21·2	20·1, 224·3	22·9	20·8, 25·1	0·201
30–39	18·3	17·5, 19·1	17·5	15·8, 19·5	18·6	16·7, 20·6	18·2	16·4, 20·1	17·7	15·8, 19·6	19·3	17·4, 21·3	18·3	16·5, 20·3	
40–49	16·2	15·5, 17·0	16·1	14·3, 18·1	16·8	15·0, 18·9	16·1	14·4, 18·1	16·9	15·1, 19·0	15·9	14·1, 17·9	15·4	13·7, 17·3	
50–59	18·0	17·2, 18·9	18·5	16·5, 20·8	17·8	15·9, 20·1	17·9	16·0, 20·1	18·9	16·9, 21·0	17·8	15·8, 19·9	17·4	15·4, 19·5	
60–69	16·2	15·4, 16·9	17·1	15·2, 18·9	16·7	14·7, 18·3	14·7	13·0, 16·4	15·4	13·5, 17·0	16·4	14·6, 18·2	17·4	15·5, 19·3	
70 and over	8·8	8·3, 9·4	8·4	7·1, 9·8	9·0	7·7, 10·6	9·5	8·1, 11·1	8·7	7·4, 10·3	8·6	7·3, 10·0	9·0	7·4, 10·2	
Education level^‡^															
Low	42·8	41·8, 43·9	43·4	40·8, 46·1	42·3	39·7, 45·0	41·9	39·3, 44·6	44·3	41·7, 46·9	42·5	39·9, 45·1	42·5	39·9, 45·1	0·169
Medium	22·2	21·4, 23·0	22·1	20·3, 24·1	23·4	21·5, 25·4	23·3	21·4, 25·3	21·9	20·0, 23·8	21·7	19·9, 23·6	20·9	19·1, 22·8	
High	35·0	34·0, 35·9	34·4	32·2, 36·7	34·3	32·1, 36·6	34·8	32·6, 37·1	33·9	31·7, 36·1	35·8	33·5, 38·1	36·6	34·3, 38·9	
Income adequacy^§^															
Very difficult/difficult	30·7	29·7, 31·7	29·7	27·3, 32·2	30·9	28·6, 33·3	30·7	28·3, 33·2	30·6	28·2, 33·0	32·3	29·9, 34·8	29·8	27·6, 32·3	0·544
Neither easy nor difficult	36·7	35·7, 37·7	37·6	35·1, 40·1	36·2	32·7, 38·7	37·2	34·7, 39·7	36·5	34·1, 39·0	35·5	33·1, 38·0	37·4	35·0, 39·9	
Easy/very easy	32·6	31·7, 33·5	32·7	30·4, 35·1	32·9	30·7, 35·2	32·1	29·9, 34·4	32·9	30·6, 35·2	32·2	30·0, 34·5	32·7	30·4, 35·1	
Nutrition knowledge^‖^															
Not at all or a little knowledgeable	37·6	36·5, 38·6	38·0	35·5, 40·6	36·5	34·1, 39·0	38·0	35·5, 40·5	36·3	33·9, 38·8	39·1	36·7, 41·7	37·8	35·3, 40·3	0·030
Somewhat knowledgeable	43·1	42·1, 44·1	42·9	40·5, 45·5	43·5	41·0, 46·0	42·3	39·8, 44·8	44·4	41·9, 47·0	41·2	38·7, 43·7	44·0	41·5, 46·6	
Very or extremely knowledgeable	19·3	18·5, 20·1	19·0	17·2, 21·1	20·0	18·1, 22·0	19·7	18·1, 22·0	19·3	17·8, 21·8	19·6	17·8, 21·6	18·2	16·8, 20·2	
Food shopping in your household^¶^															
Yes	73·1	72·1, 74·0	73·5	71·2, 75·7	72·5	70·1, 74·7	72·0	69·6, 74·2	72·1	69·8, 74·3	74·1	71·9, 76·3	74·4	72·2, 76·6	0·748
No	6·0	5·5, 6·6	5·6	4·4, 7·0	5·8	4·7, 7·2	6·1	5·0, 7·5	6·5	5·3, 8·0	6·4	5·2, 7·9	5·6	4·5, 6·9	
Share	20·9	20·0, 21·7	20·9	18·9, 23·0	21·7	19·7, 23·8	21·9	19·8, 24·0	21·4	19·4, 23·5	19·5	17·6, 21·5	20·0	18·1, 22·1	
Frequency of using nutrition information^**^															
Never/rarely	24·8	23·9, 25·8	24·1	21·9, 26·4	25·0	22·8, 27·4	24·7	22·5, 26·9	24·4	22·3, 26·8	25·5	23·3, 27·8	25·3	23·1, 27·7	0·729
Sometimes	31·8	30·9, 32·8	32·3	29·9, 34·7	32·3	29·9, 34·7	31·5	29·2, 33·9	31·2	29·2, 33·9	31·5	29·2, 33·9	32·3	29·9, 34·7	
Often/all the time	43·3	42·3, 44·4	43·7	41·1, 46·2	42·7	40·2, 45·2	43·8	41·3, 46·4	44·4	41·9, 46·9	43·0	40·5, 45·6	42·4	39·9, 44·9	
BMI category^††^															
Underweight	0·3	0·3, 0·3	0·3	0·3, 0·5	0·3	0·2, 0·4	0·3	0·2, 0·4	0·3	0·2, 0·3	0·3	0·2, 0·4	0·3	0·2, 0·4	0·131
Normal weight	34·8	33·7, 35·8	33·6	31·2, 36·0	34·1	31·8, 36·6	34·5	32·1, 36·9	35·0	32·7, 37·4	34·8	32·5, 37·3	36·4	34·0, 38·9	
Overweight	27·6	26·7, 28·5	29·3	27·0, 31·7	26·6	25·4, 29·9	27·6	25·4, 29·9	27·7	25·5, 29·9	27·3	25·1, 29·6	27·1	25·0, 29·4	
Obesity	20·7	19·8, 21·5	20·0	18·0, 22·1	22·8	20·7, 25·0	21·0	19·0, 23·1	20·4	18·4, 22·5	19·5	17·6, 21·6	20·4	18·4, 22·5	
Don’t know or no response	14·0	13·3, 14·8	13·7	11·9, 15·6	13·8	12·1, 15·7	13·9	12·1, 15·8	14·5	12·7, 16·5	15·3	13·5, 17·4	12·8	11·1, 14·7	

* Pearson *χ* ^2^ tests were calculated to determine differences by socio-demographic characteristics and ethnicity.

† Ethnic categories in each country as per census questions asked in each country: (1) Australia majority = only speaks English at home, minority = speaks a language besides English at home; (2) Canada majority = White, minority = other ethnicity; (3) Mexico majority = non-indigenous, minority = indigenous; (4) United Kingdom majority = White, minority = other ethnicity; and (5) US majority = White, minority = other ethnicity.

‡ Education level was categorised as ‘low’ (i.e. completed secondary school or less), ‘medium’ (i.e. some post-secondary qualifications) or ‘high’ (i.e. university degree or higher) according to country-specific criteria related to the highest level of formal education attained.

§ Perceived income adequacy was measured with the following question: ‘Thinking about your total monthly income, how difficult or easy is it for you to make ends meet?’ (very difficult; difficult; neither easy nor difficult; easy; very easy).

‖ Nutrition knowledge was measured with the following question: ‘How would you rate your nutrition knowledge?’ (not at all knowledgeable; a little knowledgeable; somewhat knowledgeable; very knowledgeable; extremely knowledgeable).

¶ Household shopping role was measured with the following question: ‘Do you do most of the food shopping in your household?’ (yes; no; share equally).

** Frequency of using nutrition information was measured with the following question: ‘How often do you use this type of food label when deciding to buy a food product?’ and were shown country-specific images of the Nutrition Facts information table or equivalent that is shown on the back of package in each country (never; rarely; sometimes; often; all the time).

†† BMI classification: underweight (<18·5 kg/m^2^) normal weight (18·5–24·9 kg/m^2^), overweight (25·0–29·9 kg/m^2^) and obesity (≥ 30 kg/m^2^).

Data were weighted using survey weights.

### Statistical analysis

The IFPS study sample size was powered to examine differences in nutritional outcomes between countries over time and not for each task within the survey. Post-hoc analyses indicated that with a sample size of 650 participants in each labelling condition per country and a standard deviation of 1·5, this study had an estimated 85 % power to detect a 0·25 mean difference on the 7-point Likert scale. We tested the success of randomisation of covariates by comparing variables between experimental groups using *χ* ^2^ tests.

Preliminary analyses indicated differences in label effects across countries (overall interaction effect: *X* ^2^ = 41·66, *P* = 0·003); thus, separate country models were estimated. Linear regression modelling was used to evaluate the effect of the labels on perceived healthfulness. Comparisons among label groups were made using Wald tests after running linear regression models.

We tested for possible interactions between label condition and demographic characteristics (i.e. gender, age group, income adequacy, education, nutrition knowledge, food shopping in the household, frequency of using the nutrition facts table or BMI category). For this purpose, multiplicative interactions between each demographic variable and label condition were introduced in individual country models, but only significant interactions (*P* < 0·01) were retained. In cases where demographic × label interactions were significant, associations within the demographic variables were presented, stratified by country.

Additional sensitivity analyses were performed to check the robustness of the results. Participants considering the food product as very healthy (7), healthy (6) or a little healthy (5) were classified as perceiving the product as ‘healthy’; those choosing options (4), (3), (2) or (1) were classified as perceiving the product as ‘not healthy’. We regressed this binary outcome on the experimental group.

To account for the use of several models and multiple comparisons within each, significance was set at *P* < 0·01 for regression models and test comparisons. All analyses were weighted with post-stratification sample weights constructed using a raking algorithm with population estimates from the census in each country based on age group, sex, region, ethnicity (except in Canada) and education (except in Mexico). Data analysis was performed using STATA 14.

## Results

A total of 22 140 participants were analysed (control = 3612, GDA = 3647, MTL = 3711, HSR = 3735, HIWL = 3699, HWL = 3736). No differences were observed between experimental conditions in characteristics (Table 1). Participants were evenly distributed between conditions by country, gender, age group and education level. Most (70–80 %) belonged to a majority ethnic group and were responsible for food shopping in their household, with slightly more females than males.

Stratified models showed that HIWL were the most effective label in reducing the perceived healthfulness of the fruit drink compared with the control group in all countries (range of *β*: −1·20 in the UK to −0·62 in Canada), as well as compared with the rest of the labels in Canada, Mexico, the UK and the US (Table 2).

**Table 2:** Means and regression coefficients of perceived healthfulness by label condition across countries^*^,^†^

	Australia *n* 3964				Canada *n* 4311				Mexico *n* 4057				United Kingdom *n* 5290				United States *n* 4518			
	Mean	99 % CI	*β*	99 % CI	Mean	99 % CI	*β*	99 % CI	Mean	99 % CI	*β*	99 % CI	Mean	99 % CI	*β*	99 % CI	Mean	99 % CI	*β*	99 % CI
No label control	3·53	3·38, 3·69	Reference		3·58	3·43, 3·72	Reference		4·03	3·90, 4·17	Reference		4·24	4·12, 4·36	Reference		3·89	3·74, 4·03	Reference	
GDA	3·55	3·40, 3·70	−0·04^*^	-0·28, 0·20	3·36	3·22, 3·51	−0·10^*^	-0·35, 0·15	3·97	3·83, 4·10	−0·04^*^	-0·28, 0·21	3·89	3·77, 4·03	**−0·31** ^*^	**-0·53, -0·10**	3·85	3·70, 3·99	−0·14^*^	-0·40, 0·12
MTL	3·46	3·31, 3·61	−0·14^*^	-0·39, 0·10	3·45	3·31, 3·60	−0·15^*^,^†^	-0·40, 0·11	3·76	3·62, 3·90	**−0·26** ^†^	**-0·50, -0·03**	3·79	3·66–3·92	**−0·46** ^*^,^†^	**-0·69, -0·25**	3·84	3·69, 4·00	−0·13^*^	-0·39, 0·13
HSR	2·76	2·61, 2·92	**−0·81** ^†^	**-1·07, -0·55**	3·40	3·25, 3·54	−0·19^*^,^†^	-0·44, 0·05	4·02	3·88, 4·15	0·06^*^	-0·17, 0·29	3·94	3·80–4·08	**−0·31** ^*^	**-0·53, -0·09**	3·73	3·58, 3·88	−0·18^*^,^†^	-0·44, 0·09
HWL	3·40	3·24, 3·56	−0·16^*^	-0·42, 0·09	3·22	3·08, 3·37	**−0·33** ^†^	**-0·56, -0·09**	3·61	3·47, 3·74	**−0·42** ^†^	**-0·65, -0·18**	3·68	3·56–3·81	**−0·50** ^†^	**-0·71, -0·29**	3·62	3·47, 3·78	**−0·36** ^†^	**-0·62, -0·10**
HIWL	2·69	2·53, 2·84	**−0·88** ^†^	**-1·13, -0·63**	2·91	2·76, 3·05	**−0·62**	**-0·86, -0·38**	3·18	3·02, 3·33	**−0·84**	**-1·08, -0·59**	3·00	2·86–3·14	**−1·20**	**-1·42, -0·97**	3·21	3·05, 3·37	**−0·72**	**-1·00, -0·45**

GDA, Guideline Daily Amounts; HSR, Health Star Rating; HWL, Health Warning Labels; MTL, Multiple Traffic Lights; HIWL, ‘High-in’ Warning Labels.

* Bold face indicates statistically significant (*P* < 0 01) effects compared with the control condition within each country.

† Similar superscripts within columns indicate that contrasts are not significantly different within countries (columns) (*P* < 0 01).

In Canada, Mexico and the US, HWL also led to lower perceived product healthfulness compared with the control group, but to a lesser magnitude than HIWL (range of *β*: −0·50 to −0·33). In Mexico, MTL also led to a decreased perceived healthfulness of the fruit drink compared with the control condition, with similar effects as the HWL (*β* = −0·26, 95 % CI −0·50, −0·03).

In Australia, those in the HIWL and the HSR conditions had similar decreased perceptions of product healthfulness compared with the control group (range of *β*: −0·81 to −0·88), as well as compared with the GDA, the MTL and the HWL (Table 2).

In the UK, all label conditions led to a decreased perceived healthfulness of the fruit drink compared with the control condition (Table 2). GDA, MTL and the HSR decreased the perceived product healthfulness to a similar extent in comparison with the control condition (range of *β*: −0·31 to −0·46). HWL (*β* = −0·50, 95 % CI −0·71, −0·29) had a larger effect in decreasing perceived product healthfulness than GDA and the HSR.

**Fig. 3:**
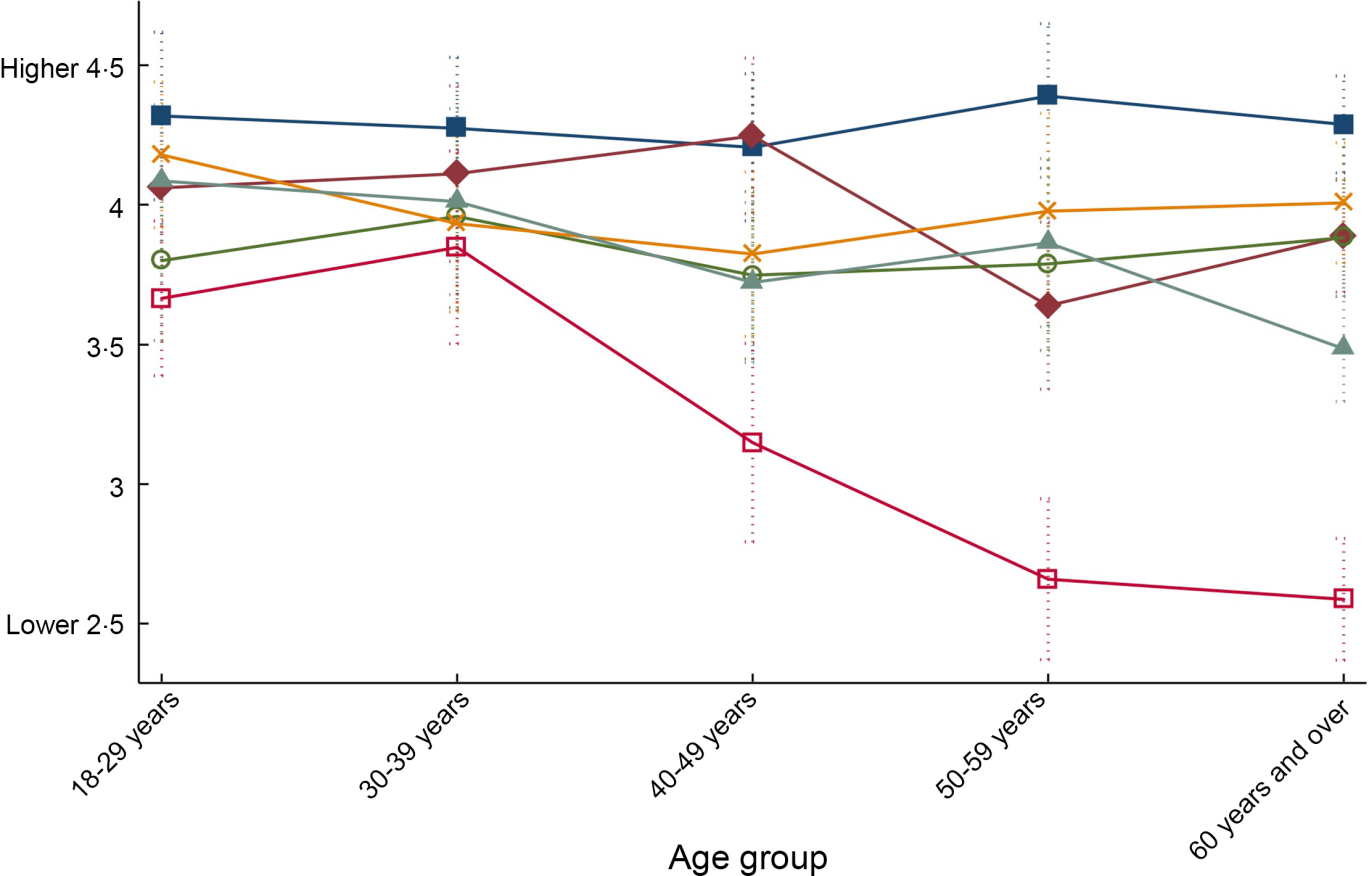
Predicted perceived healthfulness by label condition across age groups in the UK. Predictions and 95 % CI were estimated after running a linear regression model adjusted for the interaction term ‘label condition × age group’ and post-stratification sample weights. 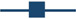, Control; 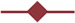, Guideline Daily Amounts (GDA); 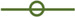, Multiple Traffic Lights (MTL); 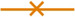, Health Star Rating (HSR); 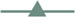, Health Warning Labels (HWL); 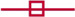, ‘High-in’ Warning Labels (HIWL)

In the UK, a statistically significant interaction between label condition and age group was observed (interaction effects *P* < 0·001) (Fig. 3). This interaction indicated that the magnitude of the impact of HIWL compared with the control condition was greater among older age groups (i.e. 40–49 years: *β* = −1·15, 95 % CI −1·64, −0·66; 50–59 years: *β* = −1·64, 95 % CI −2·04, −1·23; and 60 years and over: *β* = −1·71, 95 % CI −2·03, −1·39) than it was among those aged 18–29 years (*β* = −0·63, 95 % CI −1·02, −0·24) or 30–39 years (*β* = −0·45, 95 % CI −0·92, 0·02).

No other differences in label effects across key demographic characteristics (i.e. gender, income adequacy, education, nutrition knowledge, food shopping in the household, frequency of using the nutrition facts table or BMI category) were observed within countries.

Sensitivity analyses suggested that there were few differences in key outcomes when comparing linear and logistic regression outcomes (online supplementary material, Supplemental Table 4).

## Discussion

This study showed that the effect of FOP labels differed across countries. HIWL were the only FOP labels which consistently led participants to perceive the sweetened fruit drink as less healthy compared with the same drink without a label across all countries. In Australia only, there was a similar effect of HSR and HIWL. Other labels were effective in decreasing the perceived healthfulness of the drink within some countries only, but to a lower extent. The GDA did not exert this effect in most of the countries included in the study except the UK. In the UK, the effect of HIWL differed by age group, with greater impact among participants aged 40 and over. There were no other variations across key demographic characteristics in most countries, suggesting that different population sub-groups had similar responses to the various labelling systems.

These findings are consistent with a meta-analysis examining warning labels on sugary drinks, which showed that sugary drink warnings (HIWL or HWL) led to lower perceived product healthfulness compared with controls^(25)^. Similarly, recent studies comparing the effect of interpretive (e.g. HIWL, HSR and MTL) and reductive (i.e. GDA) FOP labelling schemes showed that interpretive labels had the greatest influence on product healthfulness perceptions^(49)^, with HIWL being the most effective among interpretive labels^(50–52)^. However, our findings are somewhat contrasting to reports by Ikonen *et al.* ^(5)^, a meta-analysis where increases in the perceived healthfulness of unhealthy products were observed for MTL and GDA, whereas no effect was reported for the HSR or HIWL. Differences may be explained by the types and relative healthfulness of products tested, and the amount of ambiguity related to their perceived healthfulness among consumers. Ikonen *et al*. included a variety of studies exploring the effects among different products, which were then re-classified as unhealthy or healthy products. However, studies suggest that larger impacts in perceived healthfulness are observed among products with intermediate healthfulness scores (e.g. breakfast cereals, yogurt, orange juice, bread), but less impact in products that people already believe are healthy (i.e. lentils and green beans) or unhealthy (i.e. potato chips)^(49–52)^. In our study, we used a sweetened fruit drink, which is often assumed to be a healthy option despite its high sugar content^(34,35)^. Given that only one type of food product was used in the experiment, one cannot assume the reported effect of labels will hold true for other food products, as has been demonstrated in other research^(5)^. Nonetheless, results build on evidence indicating that interpretive labelling schemes may be useful for decreasing perceived healthfulness of products with high content of nutrients associated with non-communicable diseases.

HIWL have become increasingly popular as a FOP label option to help consumers make healthier choices^(53)^. In contrast to most other labelling systems tested in this study, HIWL only highlight products with high amounts of critical nutrients (i.e. energy, fat, sugar and salt). Studies have shown that HIWL make excessive nutrient content and its negative health consequences more salient in consumers’ minds^(54)^. Further, evidence indicates that the black colour and the octagon shape may have stronger implicit associations with unhealthfulness^(55)^. These characteristics may explain why HIWL may be more effective messaging to communicate the idea that a product is not healthful^(5,25,26,50,56,57)^. We also observed that HIWL were more effective than HWL in communicating that the sweetened fruit drink was not healthy. Only a small number of studies have compared HIWL to HWL^(58–62)^, and more research is needed to continue answering important policy questions about how warnings can be most effectively used on food products. Future studies may also examine health warnings related to other unhealthy nutrients (e.g. Na) and for other less healthy product categories besides sugary drinks (e.g. processed meat).

In the current study, GDA had no effect on the perceived healthfulness of the product in most countries, except the UK. This finding is in line with evidence suggesting that reductive systems such as GDA, which rely on quantitative nutrient amounts, are not effective in communicating the presence of excessive amounts of critical nutrients in unhealthy foods^(49)^. As mentioned in previous reports^(3)^, these results suggest that interpretative FOP labelling systems, which incorporate elements of colour and symbolism and simplify information presented, hold more promise for conveying accurate information about product healthfulness to consumers.

Reports have also suggested that the effect of FOP labels may differ across countries. To date, most between-country studies exploring label perceptions (e.g. liking, understanding and use) or objective understanding of different FOP labels have been inconsistent, with some reporting differences across countries^(27–29)^, whereas others have not^(30,31)^. This study adds to the literature by investigating the effect of labels on perceived healthfulness of a fruit drink, finding several notable differences in the observed effect of labels across countries. It has been posited that familiarity with the labels (e.g. due to implementation of such labels and viewing labels on packages, or cultural exposure to public debates on issues of nutrition and labelling) may influence self-reported evaluations and usage intentions of labels^(29,32)^. In line with the former, the HSR was only effective in reducing perceptions of healthfulness in Australia where this policy is currently implemented on a voluntary basis; a similar effect was observed for MTL in the UK. In a broader sense, these results suggest that label effects may not be generalisable across countries and underscore the importance of producing local evidence to guide decision-making related to FOP nutrition labelling policies. Nonetheless, HIWL consistently led to lower perceived product healthfulness across all countries, suggesting that this format requires very little in the way of familiarity to be effective and may produce similar responses across high and upper-middle income countries.

The current study also examined whether the effect of labels differed across demographic characteristics. Overall, labels worked equally well across diverse populations. However, in the UK HIWL were more effective in decreasing perceived product healthfulness among older age groups than younger populations. Warning labels elicit a negative affect or perception of risk, which in turn may influence perceived product healthfulness^(26)^. Previous studies have reported greater health risk perceptions among adults and older adults compared with younger counterparts^(63,64)^, which may be explained by a greater exposure to health problems. However, the fact that label effects did not differ across income levels or nutrition knowledge, as shown in previous studies^(65,66)^, suggests that these labels are unlikely to contribute to increasing health disparities.

To our knowledge, this is the first international study comparing the effect of different FOP labels on the perceived healthfulness of a food product among countries with varying government led or mandated FOP labelling policies implemented. This study also included one Latin American country, a region which has been previously understudied. Strengths of this study include the use of a randomised design, limiting the influence of confounding from observed and unobserved factors, and a large sample size. Nonetheless, results should be interpreted within the context of several limitations. Respondents were recruited using non-probability-based sampling; therefore, the findings do not provide nationally representative estimates. However, although the descriptive statistics may not match completely with national estimates of education and BMI, the observed effects in this study provide useful information regarding the potential effects of labels across a wider population. This study focused on examining the effect of labels in perceived healthfulness using a single item measure. To expand evidence on the effectiveness of labels to communicate the relative healthfulness of products, future studies should explore the effect of labels using multiple measures and across a range of healthy products, including direct comparisons between their healthfulness and likelihood of purchase. Further, the experiment was not performed in a store; therefore, the results might have been different among some participants in a real-life situation or shopping environment. However, online food shopping is becoming increasingly common in many countries and consumers are more accustomed to rating the healthfulness of a food product when shopping online. Results of this international labelling experiment provide relevant insights for policy- and decision-makers regarding FOP labelling systems.

## Conclusions

Results indicate that warning labels are the most promising FOP labelling option to change consumer healthfulness perceptions. Specifically, HIWL may be particularly effective in helping consumers correctly identify unhealthy products with high contents of critical nutrients. Given that HIWL have been effectively implemented in several countries to date, and are compatible with international trade agreements, the current study adds to the evidence demonstrating that implementing HIWL on the front of packages is a strong policy option. The study supports the use of MTL in the UK, where this label has been implemented for more than 10 years, but has shown HIWL performed best in this country, especially among older age groups. Findings also support the consideration of the HSR for Australia, since this label performed better than the control and had a comparable effect to HIWL in this country, where this label has been implemented for more than 5 years. However, MTL were not effective outside the UK, and HSR was not effective outside Australia. Likewise, the study found little support for GDA as an option for a FOP labelling policy. Differences in label effects across countries highlight the importance of local evidence for guiding policy-making. Finally, different population sub-groups had similar responses to the various labelling systems tested in most countries, indicating FOP labels are unlikely to exacerbate disparities.

## Supplementary material

For supplementary material accompanying this paper visit https://doi.org/10.1017/S1368980021004535. click here to view supplementary material
