# Supplementary material for: Impact of front-of-pack labels on the perceived healthfulness of a sweetened fruit drink: a randomised experiment in five countries
Source: Public Health Nutr. 2021 Nov 2;25(4):1094–104. doi: 10.1017/S1368980021004535 (PMC9991717; doi:10.1017/S1368980021004535)
Supplement: Supplementary file 1 [file S1368980021004535sup001.docx]

**Supplementary Figure 1. Images with front-of-package labels displayed on screen during experimental task by country**

GDAs, Guideline Daily Amounts; HSR, Health Star Rating; HWLs, Health Warning Label; OR, Odds Ratio; MTLs, Multiple Traffic Lights; HIWLs, ‘High-in’ Warning Label.

|  | **No Label (control)** | **GDAs** | **MTLs** | **HSR** | **HWLs** | **HIWLs** |
| --- | --- | --- | --- | --- | --- | --- |
| **Australia (English)** | **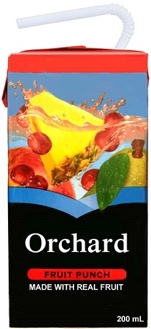** | **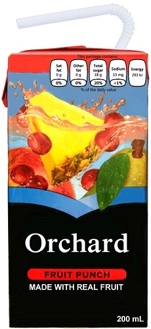** | **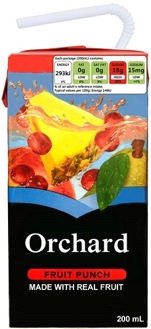** | **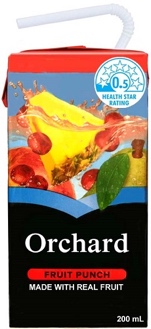** | **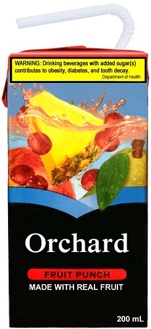** | **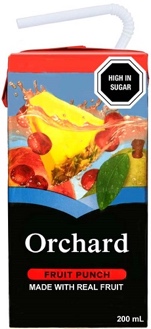** |
| **Canada (English)** | **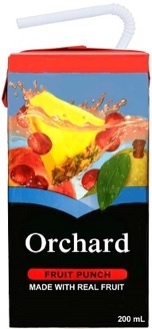** | **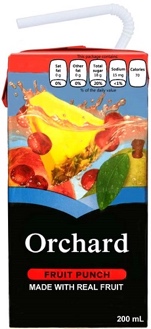** | **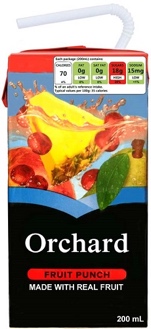** | **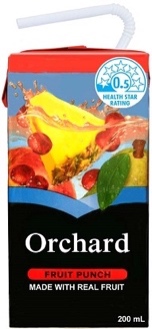** | **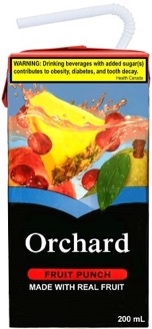** | **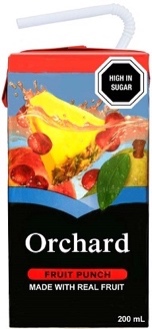** |
| **Canada**  **(French)** | **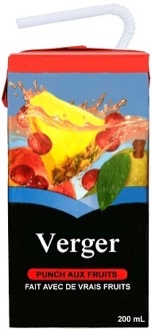** | 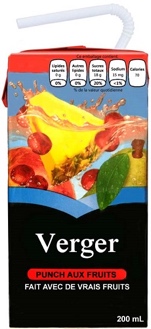 | 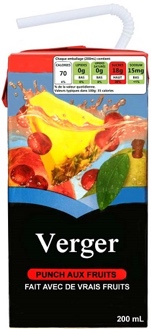 | **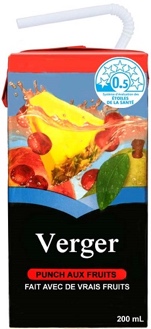** | **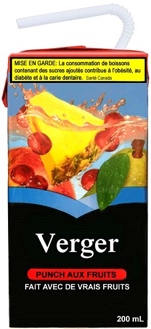** | **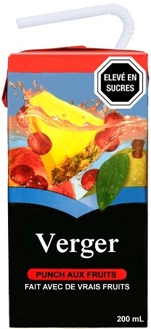** |
| **Mexico (Spanish)** | **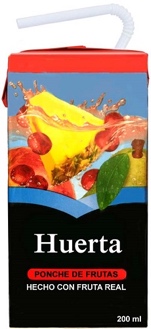** | **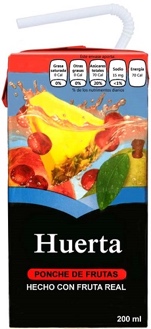** | **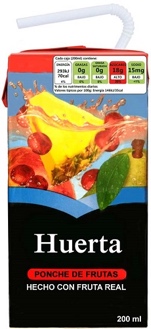** | **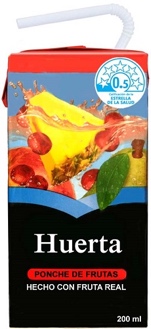** | **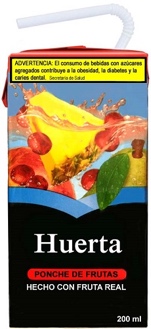** | 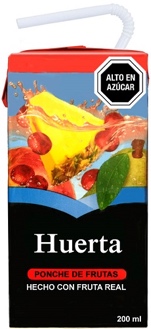 |
| **U.K.**  **(English)** | 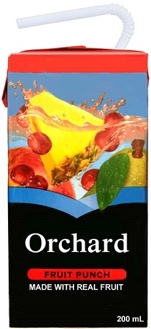 | 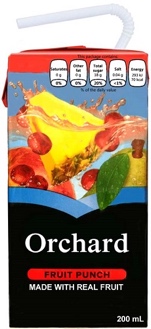 | 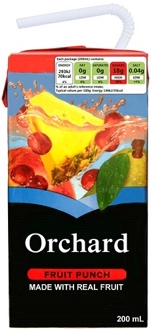 | 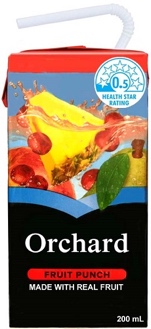 | 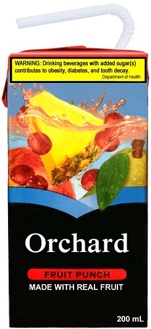 | 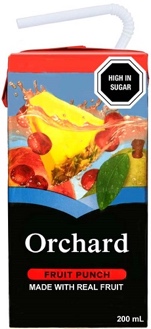 |
| **U.S. (English & Spanish)** | **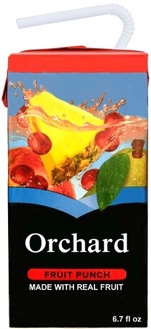** | **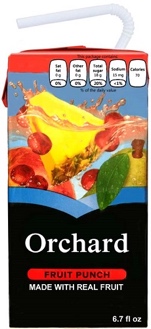** | **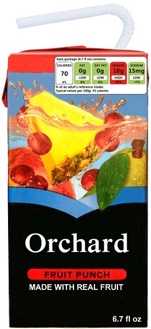** | **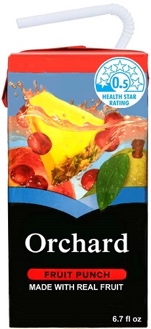** | **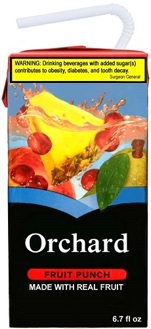** | **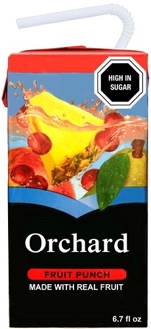** |

**Supplementary Table 1. Nutrition information used in development of product FOP labels**

| **Nutrient** | **Amount** |
| --- | --- |
| Serving Size | 200 mL (1 package) |
| Energy | 70 kcal (292.88 KJ) |
| Fat | 0 g |
| Sodium | 15 mg |
| Carbohydrates | 18 g |
| Sugars | 18 g |
| Protein | 0.1 g |
| Vitamin C | 100% of daily value |
| Concentrated fruit and vegetable percentage | 5.0% |

**Supplementary Table 2. Demographic characteristics in the analytical sample and excluded participants**

|  | **Analytic sample**  **(n=22,140)** | **Excluded**  **(n=634)** | **P value** |
| --- | --- | --- | --- |
|  | **% (95% CI)** | **% (95% CI)** |  |
| **Label condition** |  |  |  |
| No label control | 16.2 (15.6-16.8) | 19.8 (16.5-23.6) | **<0.001** |
| GDA | 16.4 (15.8-17.0) | 17.9 (14.5-21.9) |  |
| MTL | 16.8 (16.3-17.4) | 13.2 (10.2-17.0) |  |
| HSR | 17.1 (16.5-17.7) | 17.9 (14.5-22.0) |  |
| HIWL | 16.6 (16.1-17.2) | 10.9 (8.3-14.1) |  |
| HWL | 16.8 (16.3-17.4) | 20.3 (16.8-24.3) |  |
| **Country** |  |  |  |
| Australia | 17.9 (17.4-18.5) | 19.4 (16.2-23.0) | **<0.001** |
| Canada | 19.5 (18.8-20.1) | 13.2 (10.3-16.7) |  |
| Mexico | 18.3 (17.7-19.0) | 11.5 (8.8-14.9) |  |
| United Kingdom | 23.9 (23.2-24.6) | 37.4 (33.1-41.9) |  |
| United States | 20.4 (19.8-21.0) | 18.5 (15.2-22.4) |  |
| **Gender** |  |  |  |
| Male | 48.5 (47.7-49.3) | 54.4 (49.8-58.9) | **<0.001** |
| Female | 51.5 (50.7-52.3) | 45.6 (41.1-50.2) |  |
| **Ethnicity** |  |  |  |
| Majority group | 80.0 (79.3-80.6) | 81.8 (77.4-85.6) | **0.044** |
| Minority y group | 20.0 (19.4-20.7) | 18.2 (14.4-22.6) |  |
| **Age group** |  |  |  |
| 18-29 | 22.5 (21.8-23.2) | 17.6 (14.4-21.3) | **<0.001** |
| 30-39 | 18.3 (17.7-18.9) | 14.8 (12.0-18.1) |  |
| 40-49 | 16.2 (15.6-16.8) | 14.8 (11.9-18.2) |  |
| 50-59 | 18.0 (17.4-18.7) | 18.4 (14.9-22.6) |  |
| 60-69 | 16.2 (15.6-16.7) | 19.9 (16.4-23.9) |  |
| 70 and over | 8.8 (8.4-9.3) | 14.5 (11.7-17.9) |  |
| **Education level** |  |  |  |
| Low | 42.8 (42.0-43.7) | 51.8 (47.3-56.4) | **0.001** |
| Medium | 22.2 (21.6-22.8) | 21.3 (18.1-25.0) |  |
| High | 35.0 (34.3-35.7) | 26.8 (23.5-30.5) |  |
| **Income adequacy** |  |  |  |
| Very difficult / Difficult | 30.7 (29.9-31.4) | 31.0 (26.7-35.6) | 0.832 |
| Neither easy nor difficult | 36.7 (36.0-37.5) | 37.6 (33.1-42.8) |  |
| Easy/Very easy | 32.6 (31.9-33.3) | 31.5 (27.4-35.8) |  |
| **Nutrition knowledge** |  |  |  |
| Not at all or a little knowledgeable | 37.6 (36.8-38.4) | 60.3 (55.6-64.8) | **<0.001** |
| Somewhat knowledgeable | 43.1 (42.3-43.9) | 28.3 (24.4-32.4) |  |
| Very or extremely knowledgeable | 19.3 (18.7-19.9) | 11.5 (8.7-15.1) |  |
| **Food shopping in your household** |  |  | **<0.001** |
| Yes | 73.1 (72.4-73.8) | 64.3 (59.7-68.5) |  |
| No | 6.0 (5.6-6.4) | 12.4 (9.5-15.9) |  |
| Share | 20.9 (20.2-21.5) | 23.4 (19.8-27.4) |  |
| **Frequency of using the nutrient facts table** |  |  |  |
| Never/Rarely | 24.8 (24.1-25.5) | 41.6 (36.7-46.7) | **<0.001** |
| Sometimes | 31.8 (31.1-32.6) | 26.3 (22.1-30.9) |  |
| Often/All the time | 43.3 (42.6-44.1) | 32.1 (27.6-37.0) |  |
| **BMI category^b^** |  |  |  |
| Underweight | 0.3 (0.3-0.4) | 0.3 (0.2-0.5) | **<0.001** |
| Normal weight | 34.8 (34.0-35.5) | 28.1 (24.2-32.2) |  |
| Overweight | 27.6 (26.9-28.3) | 22.7 (19.1-26.8) |  |
| Obesity | 20.7 (20.0-21.3) | 18.4 (15.0-22.3) |  |
| Don’t know or no response | 14.0 (13.4-14.6) | 27.9 (24.0-32.2) |  |

**Supplementary Table 3. Missing data in the outcome across label conditions**

| **Label condition** | **N assigned** | **Excluded due to missing data in the outcome** | |
| --- | --- | --- | --- |
|  |  | **n** | **%** |
| No label control | 3,752 | 140 | 3.7 |
| Guideline Daily Amounts | 3,758 | 111 | 3.0 |
| Multiple Traffic Lights | 3,785 | 74 | 2.0 |
| Health Star Rating | 3,846 | 111 | 2.9 |
| Health Warning Label | 3,863 | 127 | 3.3 |
| ‘High-In’ Warning Label | 3,770 | 71 | 1.9 |
| No condition assigned due to technical glitch | 50 | 50 | 100 |

**Supplementary Table 4.** **Sensitivity analyses of the proportion and odds of considering the drink as healthy across countries^1,2^.**

|  | **Australia**  **n=3 964** | | **Canada**  **n=4311** | | **Mexico**  **n= 4 057** | | **United Kingdom**  **n= 5 290** | | **United States**  **n= 4 518** | |
| --- | --- | --- | --- | --- | --- | --- | --- | --- | --- | --- |
|  | **%**  **(99% CI)** | **OR**  **(99% CI)** | **%**  **(99% CI)** | **OR**  **(99% CI)** | **%**  **(99% CI)** | **OR**  **(99% CI)** | **%**  **(99% CI)** | **OR**  **(99% CI)** | **%**  **(99% CI)** | **OR**  **(99% CI)** |
| No label control | 30.3  (25.1-36.0) | 1.00 | 30.4  (25.4-35.9) | 1.00 | 35.9  (30.2-41.2) | 1.00 | 46.1  (40.9-51.3) | 1.00 | 40.1  (34.5-46.1) | 1.00 |
| GDAs | 30.5  (25.6-36.0) | 1.01 ^a^  (0.71-1.44) | 28.7  (23.4-34.6) | 0.92 ^a^  (0.64-1.33) | 35.0  (29.5-41.0) | 0.96 ^a, b^  (0.67-1.38) | 39.8  (34.8-45.1) | 0.77 ^a^  (0.57-1.05) | 36.9  (31.8-42.4) | 0.87 ^a^  (0.63-1.22) |
| MTLs | 27.5  (22.6-33.0) | 0.87 ^a^  (0.60-1.25) | 27.7  (22.8-33.2) | 0.88 ^a^  (0.61-1.25) | 30.3  (25.2-35.9) | 0.77 ^a, b, c^  (0.54-1.11) | 36.2  (31.5-41.4) | **0.67** ^a^  **(0.49-0.90)** | 37.3  (31.8-43.2) | 0.89 ^a^  (0.63-1.25) |
| HSR | 19.2  (15.1-24.0) | **0.54 ^b^**  **(0.37-0.80)** | 25.3  (20.6-30.6) | 0.78 ^a,b^  (0.54-1.12) | 38.3  (32.7-44.3) | 1.11  (0.78-1.59)^b^ | 39.2  (34.1-44.5) | 0.75 ^a^  (0.56-1.02) | 35.7  (30.5-41.3) | 0.83 ^a^  (0.59-1.16) |
| HWLs | 29.2  (24.0-34.9) | 0.95  (0.65-1.37)^a^ | 23.2  (18.8-28.4) | 0.69 ^a,b^  (0.48-1.00) | 27.5  (22.8-32.7) | **0.68** ^a,c^  **(0.47-0.97)** | 32.8  (28.0-37.9) | **0.57** ^a^  **(0.42-0.78)** | 32.5  (27.2-38.2) | 0.72 ^a,b^  (0.51-1.02) |
| HIWLs | 16.5  (12.5-21.4) | **0.45 ^b^**  **(0.30-0.69)** | 18.5  (14.1-23.8) | **0.52 ^b^**  **(0.35-0.78)** | 21.6  (17.1-26.9) | **0.49** ^c^  **(0.34-0.73)** | 23.4  (19.2-28.0) | **0.36 ^b^**  **(0.26-0.49)** | 25.7  (20.9-31.2) | **0.52** ^b^  **(0.36-0.74)** |

CI, Confidence Interval; GDAs, Guideline Daily Amounts; HSR, Health Star Rating; HWLs, Health Warning Label; OR, Odds Ratio; MTLs, Multiple Traffic Lights; HIWLs, ‘High-in’ Warning Label.

Binary outcome of perceived healthiness (1=very healthy to a little healthy, 0= neither healthy nor healthy to very unhealthy)

^1^ **Bold face** indicates statistically significant (p<0.01) effects compared to the control condition within each country.

^2^ Similar superscripts within columns indicate that contrasts are not significantly different within countries (columns) (p<0.01).
